# Supplementary material for: Validation of a Molecular Diagnostic Test for Circulating Tumor DNA by Next-Gen Sequencing
Source: Int J Mol Sci. 2023 Oct 30;24(21):15779. doi: 10.3390/ijms242115779 (PMC10648112; doi:10.3390/ijms242115779)
Supplement: Supplementary file 1 [file ijms-24-15779-s001.zip › ijms-2681168-supplementary.pdf]

A)

PGDx elio™ plasma resolve Gene Panel					
**Sequence Mutation Analysis (33 Genes)**					
AKT1	BRCA1	CSF1R	HRAS	NTRK1	RET
ALK	BRCA2	EGFR	KIT	PDGFRA	ROS1
APC	BRIP1	ERBB2	KRAS	PIK3CA	TP53
ARID1A	CCND1	EZH2	MET	POLD1	
ATM	CD274	FGFR1	MYC	POLE	
BRAF	CDH1	FGFR2	NRAS	RAF1	
Amplifications (8)					
CCND1	EGFR	FGFR2	MET		
CD274	ERBB2	KIT	MYC		
Translocations (5)					
ALK	FGFR2	NTRK1	RET	ROS1	

Suppl. Table S1. PGDx EPR panel.

Gene ID	COSMIC Identifier	Mutation Type	HGVS Nomenclature	Amino Acid Change
AKT1	COSM33765	Substitution	c.49G>A	p.E17K
APC	COSM13127	Substitution	c.4348C>T	p.R1450*
APC	COSM18561	Insertion in HP 7N	c.4666_4667insA	p.T1556fs*3
ATM	COSM21924	Deletion	c.1058_1059delGT	p.C353fs*5
BRAF	COSM476	Substitution	c.1799T>A	p.V600E
CTNNB1	COSM5664	Substitution	c.121A>G	p.T41A
EGFR	COSM6224	SNV in 3N	c.2573T>G	p.L858R
EGFR	COSM12378	Insertion	c.2310_2311insGGT	p.D770_N771insG
EGFR	COSM6225	Deletion	c.2236_2250del15	p.E746_A750delELREA
EGFR	COSM6240	Substitution	c.2369C>T	p.T790M
ERBB2	COSM682/20959	Insertion	c.2324_2325ins12	p.A775_G776insYVMA
FGFR3	COSM715	Substitution	c.746C>G	p.S249C
FLT3	COSM783	Substitution	c.2503G>T	p.D835Y
FOXL2	COSM33661	Substitution	c.402C>G	p.C134W
GNA11	COSM52969	Substitution	c.626A>T	p.Q209L
GNAQ	COSM28758	SNV in HP 3N	c.626A>C	p.Q209P
GNAS	COSM27887	Substitution	c.601C>T	p.R201C
IDH1	COSM28747	Substitution	c.394C>T	p.R132C
JAK2	COSM12600	SNV in HP 3N	c.1849G>T	p.V617F
KIT	COSM1314	Substitution	c.2447A>T	p.D816V
KRAS	COSM521	Substitution	c.35G>A	p.G12D
MPL	COSM18918	Substitution	c.1544G>T	p.W515L
NCOA4-RET	N/A	Gene Fusion (DNA)	NCOA4(NC_000010.10):r.1_1014+1312_RET(NC_000010.10):r.2327-1437_5659	N/A
NPM1	COSM17559	Insertion	c.863_864insTCTG	p.W288fs*12
NRAS/CSDE1	COSM584	Substitution	c.182A>G	p.Q61R
PDGFRA	COSM736	Substitution	c.2525A>T	p.D842V
PDGFRA	COSM28053	Insertion	c.1694_1695insA	p.S566fs*6
PIK3CA	COSM763	Substitution	c.1633G>A	p.E545K
PIK3CA	COSM12464	Insertion	c.3204_3205insA	p.N1068fs*4
PIK3CA	COSM775	Substitution	c.3140A>G	p.H1047R
PTEN	COSM4986	Insertion	c.741_742insA	p.P248fs*5
PTEN	COSM5809	Deletion 6N > 5N	c.800delA	p.K267fs*9
RET	COSM965	Substitution	c.2753T>C	p.M918T
SMAD4	COSM14105	Insertion	c.1394_1395insT	p.A466fs*28
TP53	COSM10648	Substitution	c.524G>A	p.R175H
TP53	COSM10660	Substitution	c.818G>A	p.R273H
TP53	COSM10662	Substitution	c.743G>A	p.R248Q
TP53	COSM6530	Deletion	c.723delC	p.C242fs*5
TP53	COSM18610	Deletion 5N >4N	c.263delC	p.S90fs*33
TPR-ALK	N/A	Gene Fusion (DNA)	TPR(NC_000001.10):r.1_2185+246_ALK(NC_000002.11):r.4125-550_6265	N/A

Suppl. Table S2. Variants in Seraseq ctDNA Reference Material version 2. Highlighted in yellow, variants that can be potentially detected with PGDx EPR panel.

		30 ng 2%	30 ng 2%	30 ng 2%	30ng 1%	30ng 1%	30ng 1%	30 ng 0.50%	30 ng 0.50%	30 ng 0.50%	30 ng 0.25%	30 ng 0.25%	30 ng 0.25%	30 ng 0.125	30 ng 0.125	30 ng 0.125
Variants																
AKT1 c.49G>A; p. E17K	Path	1.83	1.33	1.8	1.08	0.9	0.67	0.48	0.82	0.87	0.55	NO	NO	NO	NO	NO
APC c.4348C>T; p.R1450*	Path	1.47	1.2	1.86	0.75	0.86	1.06	0.64	NO	NO	NO	NO	NO	NO	NO	NO
BRAF c.1799T>A; V600E	Path	1.78	2.02	2.51	0.9	1.04	0.53	0.29	0.52	0.86	0.22	0.17	NO	0.31	0.27	NO
EGFR c.2369C>p.T790M	Path	2.16	1.78	2.06	1.08	1.04	0.8	0.34	0.44	0.72	0.26	0.19	0.23	NO	NO	NO
EGFR c.2573T>G; p.L858R	Path	2.42	2.09	2.14	1.4	1.18	1.02	0.95	0.58	0.52	0.25	0.37	0.24	NO	NO	0.26
KIT c.2447A>T; p.D816V	Path	2.32	1.33	2.06	0.94	0.76	0.91	0.29	0.52	0.25	0.22	NO	0.21	0.12	NO	0.15
KRAS c.35G>A; p.G12D	Path	1.77	1.1	1.58	1.42	0.76	0.7	0.5	0.39	0.44	0.25	0.16	0.2	0.17	0.11	0.15
NRAS c.182A>G; p.Q61R	Path	2.23	2.3	2.4	1.81	0.99	1.01	0.41	0.96	0.43	0.11	0.24	0.12	0.31	0.42	0.27
PDGFRA c.2525A>T; p.D842V	Path	2.24	1.79	1.71	1.07	0.95	0.91	0.42	0.41	0.26	0.26	0.33	0.25	0.32	0.17	NO
PIK3CA c.1633G>A; p.E545K	Path	1.79	NO	1.45	0.9	0.66	0.58	0.3	NO	NO	0.19	NO	0.35	NO	NO	NO
PIK3CA c.3140A>G; p.H1047R	Path	2.24	1.83	1.52	1.02	0.89	0.7	0.45	0.44	0.58	0.18	0.11	0.31	0.17	0.16	NO
RET c.2753T>C; p.M918T	Path	2.46	1.49	1.8	0.89	0.77	0.46	0.7	0.45	0.34	0.39	0.11	NO	0.11	0.15	NO
TP53 c.524G>A; p.R175H	Path	2.44	1.59	2.44	1.37	0.91	1.17	0.46	0.63	0.44	NO	0.37	NO	NO	NO	NO
TP53 c.743G>A; R248Q	Path	2.37	1.16	1.62	0.92	0.76	1.02	0.57	0.56	0.39	NO	NO	NO	NO	NO	NO
TP53 c.818G>A; p.R273H	Path	2.39	1.87	1.88	1.35	1.4	1.04	0.75	0.61	0.31	NO	0.33	NO	NO	NO	NO
APC c.4666_4667insA; p.T1556fs*3	Path	NO	NO	NO	NO	NO	NO	NO	NO	NO	NO	NO	NO	NO	NO	NO
ATM 1057_1058delTG; p. C353fs*5	Path	1.59	1.15	1.76	NO	0.9	0.89	0.58	0.61	NO	NO	NO	NO	NO	NO	NO
2236_2250delGAATTAAGAGAAGCA; p.E746_A750delELREA	Path	2.33	1.82	2.2	0.89	1.26	1.26	0.57	0.58	0.73	0.26	0.35	0.34	NO	0.16	0.2
EGFR c.2310_2311 insGGT; p.D770_N771 insG	Path	2.13	1.7	1.74	1.05	1.1	0.8	0.47	0.39	0.6	0.26	0.19	0.23	0.11	NO	NO
ERBB2 c.2310_2311insGCATACGTGATG; E770_A771insAYVM	Path	2.02	1.81	1.19	0.97	0.91	0.66	0.56	0.33	0.51	NO	0.25	0.11	0.11	NO	NO
PDGFRA c.1694_1695insA; p.S566fs*6	Unc.sig	2.64	1.9	2.39	1.13	1.71	1.17	0.84	0.58	0.82	NO	NO	NO	NO	NO	NO
PIK3CAc.3204_3205insA; p.1069fs*	Unc.sig.	1.71	1.82	1.61	0.89	1.43	0.66	NO	NO	0.67	NO	NO	NO	NO	NO	NO
TP53 c.723delC; p.C242fs*5	Path	2.31	1.27	1.51	0.92	0.76	0.93	0.56	0.53	0.36	0.32	0.33	NO	NO	NO	NO
TP53 c.263delC; p.S90fs*33	Path	NO	NO	NO	NO	NO	NO	NO	NO	NO	NO	NO	NO	NO	NO	NO
False positives		Two	One	Two	Two	One	None	One	None	None	None	None	None	None	None	None
Library #		118	217	223	120	218	219	122	Yinfei 1	220	213	215	135	139	Yinfei 5	221

Suppl. Table S3. Variants detected by PGDx EPR using 30 ng of Seraseq controls for library preparation. A total of 15 libraries were prepared using 30 ng of the Seraseq Ref. Mat. V2. controls: 3 libraries for each Seraseq controls (2%, 1%, 0.5%, 0.25% and 0.125%). The variant allele frequency (VAF) detected by PGDx for each mutation is indicated in each case. The number of false positives (FP) variants detected in each library are also indicated at the bottom of the table. Highlighted in yellow (NO) indicates variants not detected by the PGDx EPR assay. Path, pathogenic; Unc.sig., variants of uncertain significance.

		40 ng	40 ng	40 ng	40 ng	40 ng	40 ng	40 ng	40 ng	40 ng	40 ng	40 ng	40 ng	40 ng	40 ng	40 ng	40 ng	40 ng	40 ng
		2%	2%	2%	1%	1%	1%	0.50%	0.50%	0.50%	0.50%	0.50%	0.25%	0.25%	0.25%	0.125	0.125	0.125	0.125
AKT1 c.49G>A; p. E17K	Path	1.81	1.66	1.38	0.78	1.14	1.19	0.66	0.55	0.57	0.61	0.45	0.35	NO	0.31	NO	NO	NO	NO
APC c.4348C>T; p.R1450*	Path	1.6	1.57	1.57	0.67	1.01	1.06	0.38	0.41	0.36	0.5	NO	NO	NO	NO	NO	NO	NO	NO
BRAF c.1799T>A; V600E	Path	1.93	2.41	1.68	1.16	0.96	1.5	0.48	0.43	0.53	0.65	0.18	0.23	0.2	NO	NO	NO	NO	NO
EGFR c.2369C>T; p.T790M	Path	2	1.84	2.46	0.98	1.08	1.3	0.52	0.46	0.4	0.49	0.46	0.42	0.26	0.3	NO	0.21	0.17	0.21
EGFR c.2573T>G; p.L858R	Path	2.19	1.55	1.47	1.19	0.91	1.19	0.61	0.45	0.56	0.42	0.71	0.27	0.19	0.16	0.17	NO	0.11	0.19
KIT c.2447A>T; p.D816V	Path	2.16	1.72	1.38	0.75	0.49	1.17	0.29	0.74	0.23	0.32	0.32	0.31	0.15	0.48	0.19	NO	0.22	0.12
KRAS c.35G>A; p.G12D	Path	1.75	1.35	1.59	1.01	1.04	0.89	0.35	0.72	0.67	0.32	0.39	0.2	0.23	0.13	0.23	NO	NO	NO
NRAS c.182A>G; p.Q61R	Path	2.11	1.69	2.27	1.09	1.22	1	0.64	0.46	0.49	0.81	0.17	0.16	0.19	0.14	0.16	0.18	0.24	0.31
PDGFRA c.2525A>T; p.D842V	Path	1.88	2	1.88	0.94	0.78	1.16	0.41	0.52	0.48	0.43	0.54	0.28	0.2	0.36	NO	0.18	0.19	NO
PIK3CA c.1633G>A; p.E545K	Path	1.75	0.8	1.11	1.15	0.37	0.78	0.51	NO	0.78	NO	0.55	0.15	0.33	0.35	NO	NO	NO	NO
PIK3CA c.3140A>G; p.H1047R	Path	1.6	1.46	1.55	0.98	0.78	0.77	0.36	0.43	0.47	0.33	0.2	0.21	0.15	0.29	0.12	NO	NO	0.13
RET c.2753T>C; p.M918T	Path	1.75	1.7	1.91	0.87	0.78	1.04	0.37	0.42	0.62	0.48	0.43	0.35	0.22	0.17	0.13	NO	NO	0.12
TP53 c.524G>A; p.R175H	Path	2.09	2.22	2.33	1.18	1.42	1.06	0.45	0.55	0.46	0.53	NO	NO	NO	0.4	NO	NO	NO	NO
TP53 c.743G>A; R248Q	Path	2.3	1.87	1.37	0.99	0.93	0.94	0.65	0.45	0.42	0.47	0.48	0.31	NO	0.36	NO	NO	NO	NO
TP53 c.818G>A; p.R273H	Path	2.53	1.03	1.89	0.99	0.8	1.1	0.45	0.45	0.42	0.58	NO	NO	NO	0.53	NO	NO	NO	NO
APC c.4666_4667insA; p.T1556fs*3	Path	NO	NO	NO	NO	NO	NO	NO	NO	NO	NO	NO	NO	NO	NO	NO	NO	NO	NO
ATM 1057_1058delTG; p. C353fs*5	Path	1.56	0.96	1.7	1.28	0.68	0.66	NO	NO	NO	0.51	NO	NO	NO	NO	NO	NO	NO	NO
EGFR 2236_2250delGAATTAAGAGAAGCA; p.E746_A750delELREA	Path	2.29	1.91	2.25	1.38	1.22	1.37	0.48	0.59	0.57	0.32	0.91	0.21	0.2	0.39	0.2	0.12	NO	0.25
EGFR c.2310_2311 insGGT; p.D770_N771 insG	Path	1.83	1.85	2.14	0.97	0.93	1.21	0.42	0.44	0.41	0.36	0.61	0.32	0.25	0.25	NO	0.12	0.12	0.14
ERBB2 2310_2311insGCATACGTGATG; E770_A771insAYVM	Path	2.07	2.19	1.9	0.94	0.87	0.91	0.51	0.53	0.41	0.43	0.66	0.18	NO	0.18	0.14	NO	NO	NO
PDGFRA c.1694_1695insA; p.S566fs*6	Unc.sig	2.78	2.95	2.75	1.09	1.29	1.61	NO	0.61	0.62	0.7	0.52	NO	NO	0.51	NO	NO	NO	NO
PIK3CAc.3204_3205insA; p.*1069fs*	Unc.sig.	1.42	0.99	1.34	1.07	0.82	0.65	NO	0.53	NO	NO	NO	NO	NO	NO	NO	NO	NO	NO
TP53 c.723delC; p.C242fs*5	Path	2.3	1.94	1.32	0.88	0.87	0.97	0.6	0.39	0.38	0.42	0.46	NO	NO	0.35	NO	NO	NO	NO
TP53 c.263delC; p.S90fs*33	Path	NO	NO	NO	NO	NO	NO	NO	NO	NO	NO	NO	NO	NO	NO	NO	NO	NO	NO
		One	One	None	None	None	None	None	None	None	None	Three	One	None	None	None	Two	None	None
		119	YF-VAF2-40ng	207	121	YT-LIB22-VAF1	YT-LIB23-	123	131	Yinfei2	YT-LIB21-	YT-LIB19-	136	209	210	140	143c	YT-LIB17-VA	YT-LIB18-

Suppl. Table S4. Variants detected by PGDx EPR using 40 ng of Seraseq controls for library preparation. A total of 18 libraries were prepared using 40 ng Seraseq Ref. Mat. V.2 controls: 3 libraries with 2% control, 3 libraries with 1% control, 5 libraries with 0.5% control, 3 libraries with 0.250% control, and 4 libraries with 0.125% control. The variant allele frequency (VAF) detected by PGDx for each mutation is indicated in each case. The number of false positives variants detected in each library are also indicated at the bottom of the table. Highlighted in yellow (NO) indicates variants not detected by the PGDx EPR assay. Path, pathogenic; Unc.sig., variants of uncertain significance.

		50 ng	50 ng	50 ng	50 ng	50 ng	50 ng	50 ng	50 ng	50 ng	50 ng	50 ng	50 ng	50 ng	50 ng	50 ng
		2%	2%	2%	1%	1%	1%	0.50%	0.50%	0.50%	0.25%	0.25%	0.25%	0.125	0.125	0.125
AKT1 c.49G>A; p. E17K	Path	1.45	1.77	1.63	0.92	0.87	0.65	0.43	0.36	0.37	0.45	NO	NO	NO	NO	NO
APC c.4348C>T; p.R1450*	Path	1.28	1.6	1.36	0.6	0.84	0.89	0.5	0.34	0.55	NO	NO	NO	NO	NO	NO
BRAF c.1799T>A; V600E	Path	1.74	1.79	1.38	0.45	0.94	0.85	0.45	0.37	0.28	0.16	0.26	0.24	0.15	0.24	0.25
EGFR c.2369C>T; p.T790M	Path	2.3	1.95	2.1	1.06	1.15	1.01	0.28	0.46	0.37	0.35	0.38	0.28	NO	NO	0.16
EGFR c.2573T>G; p.L858R	Path	2.21	2.2	2.23	0.95	1.03	1.2	0.43	0.26	0.38	0.4	0.3	0.31	NO	0.18	NO
KIT c.2447A>T; p.D816V	Path	1.33	1.86	1.66	0.88	0.66	0.85	0.42	0.3	0.24	0.15	0.15	0.13	0.25	NO	0.11
KRAS c.35G>A; p.G12D	Path	1.6	1.89	1.24	0.66	1.06	0.75	0.4	0.34	0.51	NO	0.16	0.32	0.15	NO	NO
NRAS c.182A>G; p.Q61R	Path	2.35	2.03	2	0.99	1.63	1.17	0.37	0.59	0.53	0.11	0.37	0.27	0.23	0.14	0.16
PDGFRA c.2525A>T; p.D842V	Path	1.98	2	2.24	1.06	1.1	0.78	0.42	0.4	0.41	0.38	0.29	0.14	0.16	NO	0.13
PIK3CA c.1633G>A; p.E545K	Path	1.33	1.89	1.43	0.9	1.06	1.45	0.44	0.61	NO	0.42	0.66	0.3	NO	NO	NO
PIK3CA c.3140A>G; p.H1047R	Path	1.96	1.75	1.77	0.81	0.88	0.74	0.54	0.29	0.64	0.1	0.34	0.16	0.24	NO	NO
RET c.2753T>C; p.M918T	Path	1.72	1.62	1.37	0.83	0.82	0.92	0.55	0.36	0.44	0.27	0.31	0.35	0.11	NO	0.25
TP53 c.524G>A; p.R175H	Path	1.95	2	1.95	1.13	1.05	1.4	0.43	0.59	0.53	0.56	0.39	NO	NO	NO	NO
TP53 c.743G>A; R248Q	Path	1.68	1.79	1.47	0.79	0.78	0.85	0.6	0.39	0.4	NO	NO	NO	NO	NO	NO
TP53 c.818G>A; p.R273H	Path	1.66	2.04	1.65	0.77	0.99	1.16	0.43	0.34	NO	0.4	NO	NO	NO	NO	NO
APC c.4666_4667insA; p.T1556fs*3	Path	NO	NO	NO	NO	NO	NO	NO	NO	NO	NO	NO	NO	NO	NO	NO
ATM c.1057_1058delTG; p. C353fs*5	Path	1.45	2.02	1.39	NO	0.99	NO	0.65	0.54	NO	NO	NO	NO	NO	NO	NO
EGFR c.2236_2250delGAATTAAGAGAAGCA; p.E746_A750delELREA	Path	2	2.18	2.29	1.07	1.15	1.24	0.38	0.52	0.37	0.19	0.2	0.21	NO	0.2	NO
EGFR c.2310_2311 insGGT; p.D770_N771 insG	Path	2.29	1.77	1.84	1.11	1.03	0.78	0.24	0.42	0.37	0.32	0.26	0.16	NO	NO	0.13
ERBB2 c.2310_2311insGCATACGTGATG; E770_A771insAYVM	Path	1.33	1.09	2.17	0.95	0.9	0.99	0.42	0.54	0.38	0.11	0.17	0.24	0.12	0.16	0.13
PDGFRA c.1694_1695insA; p.S566fs*6	Unc.sig	2.42	2.05	2.65	1.34	1.26	1.22	NO	NO	NO	NO	NO	NO	NO	NO	NO
PIK3CAc.3204_3205insA; p.*1069fs*	Unc.sig.	1.89	1.9	1.65	0.78	0.82	0.71	0.76	NO	0.59	NO	NO	NO	NO	NO	NO
TP53 c.723delC; p.C242fs*5	Path	1.63	1.68	1.47	0.72	0.77	0.8	0.57	0.42	0.39	NO	NO	NO	NO	NO	NO
TP53 c.263delC; p.S90fs*33	Path	NO	NO	NO	NO	NO	NO	NO	NO	NO	NO	NO	NO	NO	NO	NO
False positives		One	One	None	None	None	None	None	Two	None	None	None	None	One	One	One
Library #		127	224	239	129	225	240	124	132	Yinfei3	137	226	212	141	144c	Yinfei7

Suppl. Table S5. Variants detected by the PGDx EPR assay using 50 ng of Seraseq controls for library preparation. A total of 15 libraries were prepared. The variant allele frequency (VAF) detected by PGDx for each mutation is indicated in each case. The number of false positives variants detected in each library are also indicated at the bottom of the table. Highlighted in yellow (NO) indicates variants not detected by the PGDx EPR assay. Path, pathogenic; Unc.sig., variants of uncertain significance.

		60 ng	60 ng	60 ng	60 ng	60 ng	60 ng	60 ng	60 ng	60 ng	60 ng	60 ng	60 ng	60 ng	60 ng	60 ng
		2%	2%	2%	1%	1%	1%	0.50%	0.50%	0.50%	0.25%	0.25%	0.25%	0.125	0.125	0.125
AKT1 c.49G>A; p. E17K	Path	1.45	1.72	1.82	0.95	1.26	1.15	0.54	0.35	0.49	NO	0.41	NO	NO	NO	NO
APC c.4348C>T; p.R1450*	Path	1.5	1.43	1.67	0.72	0.84	0.85	0.31	0.59	0.51	NO	NO	NO	NO	NO	NO
BRAF c.1799T>A; V600E	Path	2.37	1.74	1.81	0.61	0.9	1.02	0.26	0.17	0.31	0.2	0.21	0.17	NO	NO	NO
EGFR c.2369C>T; p.T790M	Path	1.99	2.08	2.31	1.12	1.31	1.08	0.59	0.45	0.63	0.23	0.36	NO	NO	0.17	0.18
EGFR c.2573T>G; p.L858R	Path	1.74	1.68	1.92	0.9	0.95	1.5	0.53	0.53	0.29	0.24	0.31	0.25	0.23	0.17	0.11
KIT c.2447A>T; p.D816V	Path	1.75	1.82	1.45	1.02	0.48	0.86	0.48	0.36	0.67	0.24	0.42	0.18	NO	NO	0.23
KRAS c.35G>A; p.G12D	Path	1.88	1.97	1.62	1.2	1.11	0.9	0.46	0.27	0.56	NO	0.22	0.27	NO	0.12	0.25
NRAS c.182A>G; p.Q61R	Path	2.1	2.54	2.18	1.19	1.43	1.33	0.57	0.64	0.31	0.15	0.3	0.15	0.19	0.17	0.12
PDGFRA c.2525A>T; p.D842V	Path	2.04	1.89	1.87	0.84	0.77	0.75	0.54	0.3	0.63	0.31	0.46	0.28	0.12	NO	0.1
PIK3CA c.1633G>A; p.E545K	Path	1.58	1.66	1.48	0.28	0.67	0.93	0.31	NO	0.18	0.23	NO	0.22	NO	NO	NO
PIK3CA c.3140A>G; p.H1047R	Path	1.62	1.92	1.34	1.1	0.97	1.17	0.56	0.36	0.66	0.26	0.2	0.35	NO	NO	0.17
RET c.2753T>C; p.M918T	Path	1.61	1.92	1.92	1.07	0.72	0.78	0.4	0.5	0.63	0.22	0.29	0.17	NO	NO	0.1
TP53 c.524G>A; p.R175H	Path	2.29	2.61	2.24	1.26	1.14	1.01	0.48	0.59	0.51	0.3	NO	0.32	NO	NO	NO
TP53 c.743G>A; R248Q	Path	1.52	1.67	1.54	0.52	0.72	0.87	0.38	0.43	0.42	NO	NO	0.3	NO	NO	NO
TP53 c.818G>A; p.R273H	Path	1.82	1.78	1.57	1.09	0.98	0.92	NO	0.44	NO	NO	NO	NO	NO	NO	NO
APC c.4666_4667insA; p.T1556fs*3	Path	NO	NO	NO	NO	NO	NO	NO	NO	NO	NO	NO	NO	NO	NO	NO
ATM c.1057_1058delTG; p. C353fs*5	Path	1.85	1.68	1.21	NO	0.65	0.83	NO	NO	NO	NO	NO	NO	NO	NO	NO
EGFR c.2236_2250delGAATTAAGAGAAGCA; p.E746_A750delELREA	Path	2.23	2.28	2.09	1.45	0.87	1.56	0.51	0.6	0.82	0.2	0.27	0.27	0.13	NO	0.17
EGFR c.2310_2311 insGGT; p.D770_N771 insG	Path	1.73	1.78	2.09	0.93	1.12	1.09	0.52	0.4	0.56	0.2	0.27	0.18	NO	NO	NO
ERBB2 c.2310_2311insGCATACGTGATG; E770_A771insAYVM	Path	2.08	2.09	2.11	1.11	1.06	1.29	0.48	0.42	0.63	0.15	0.25	0.24	0.16	0.14	0.13
PDGFRA c.1694_1695insA; p.S566fs*6	Unc.sig	2.21	2.61	2.06	1.39	1.22	0.99	0.53	0.52	0.76	NO	NO	NO	NO	NO	NO
PIK3CAc.3204_3205insA; p.*1069fs*	Unc.sig.	1.81	1.75	1.18	0.92	0.96	1.04	NO	NO	0.81	NO	NO	NO	NO	NO	NO
TP53 c.723delC; p.C242fs*5	Path	1.43	1.61	1.46	0.54	0.68	0.89	0.41	0.44	0.4	NO	NO	NO	NO	NO	NO
TP53 c.263delC; p.S90fs*33	Path	NO	NO	NO	NO	NO	NO	NO	NO	NO	NO	NO	NO	NO	NO	NO
False positives		None	One	One	None	One	One	None	None	None	None	None	None	None	None	None
Library #		128	232	233	130	234	235	133	Yinfei 4	236	138	237	241	145c	242	243

Suppl. Table S6. **Variants detected by PGDx EPR using 60 ng of Seraseq controls for library preparation.** A total of 15 libraries were prepared. The variant allele frequency (VAF) detected by PGDx for each mutation is indicated in each case. The number of false positives variants detected in each library are also indicated at the bottom of the table. Highlighted in yellow (NO) indicates variants not detected by the PGDx EPR assay. Path, pathogenic; Unc.sig., variants of uncertain significance

		30 ng	30 ng	30 ng	40 ng	40 ng	40 ng	40 ng	50 ng	50 ng	50 ng	60 ng	60 ng	60 ng
AKT1 c.49G>A; p. E17K	Path	NO	NO	NO	NO	NO	NO	NO	NO	NO	NO	NO	NO	NO
APC c.4348C>T; p.R1450*	Path	NO	NO	NO	NO	NO	NO	NO	NO	NO	NO	NO	NO	NO
BRAF c.1799T>A; V600E	Path	NO	NO	NO	NO	NO	NO	NO	NO	NO	NO	NO	NO	NO
EGFR c.2369C>p.T790M	Path	NO	NO	NO	NO	NO	NO	NO	NO	NO	NO	NO	NO	NO
EGFR c.2573T>G; p.L858R	Path	NO	NO	NO	NO	NO	NO	NO	NO	NO	NO	NO	NO	NO
KIT c.2447A>T; p.D816V	Path	NO	NO	NO	NO	NO	NO	NO	NO	NO	NO	NO	NO	NO
KRAS c.35G>A; p.G12D	Path	NO	NO	NO	NO	NO	NO	NO	NO	NO	NO	NO	NO	NO
NRAS c.182A>G; p.Q61R	Path	NO	NO	NO	NO	NO	NO	NO	NO	NO	NO	NO	NO	NO
PDGFRA c.2525A>T; p.D842V	Path	NO	NO	NO	NO	NO	NO	NO	NO	NO	NO	NO	NO	NO
PIK3CA c.1633G>A; p.E545K	Path	NO	NO	NO	NO	NO	NO	NO	NO	NO	NO	NO	NO	NO
PIK3CA c.3140A>G; p.H1047R	Path	NO	NO	NO	NO	NO	NO	NO	NO	NO	NO	NO	NO	NO
RET c.2753T>C; p.M918T	Path	NO	NO	NO	NO	NO	NO	NO	NO	NO	NO	NO	NO	NO
TP53 c.524G>A; p.R175H	Path	NO	NO	NO	NO	NO	NO	NO	NO	NO	NO	NO	NO	NO
TP53 c.743G>A; R248Q	Path	NO	NO	NO	NO	NO	NO	NO	NO	NO	NO	NO	NO	NO
TP53 c.818G>A; p.R273H	Path	NO	NO	NO	NO	NO	NO	NO	NO	NO	NO	NO	NO	NO
APC c.4666_4667insA; p.T1556fs*3	Path	NO	NO	NO	NO	NO	NO	NO	NO	NO	NO	NO	NO	NO
ATM c. 1057_1058delTG; p. C353fs*5	Path	NO	NO	NO	NO	NO	NO	NO	NO	NO	NO	NO	NO	NO
EGFR c.2236_2250delGAATTAAGAGAAGCA; p.E746_A750delELREA	Path	NO	NO	NO	NO	NO	NO	NO	NO	NO	NO	NO	NO	NO
EGFR c.2310_2311 insGGT; p.D770_N771 insG	Path	NO	NO	NO	NO	NO	NO	NO	NO	NO	NO	NO	NO	NO
ERBB2 c.2310_2311insGCATACGTGATG; E770_A771insAYVM	Path	NO	NO	NO	NO	NO	NO	NO	NO	NO	NO	NO	NO	NO
PDGFRA c.1694_1695insA; p.S566fs*6	Unc.sig	NO	NO	NO	NO	NO	NO	NO	NO	NO	NO	NO	NO	NO
PIK3CAc.3204_3205insA; p. *1069fs*	Unc.sig.	NO	NO	NO	NO	NO	NO	NO	NO	NO	NO	NO	NO	NO
TP53 c.723delC; p.C242fs*5	Path	NO	NO	NO	NO	NO	NO	NO	NO	NO	NO	NO	NO	NO
TP53 c.263delC; p.S90fs*33	Path	NO	NO	NO	NO	NO	NO	NO	NO	NO	NO	NO	NO	NO
False positives		Three	None	Two	One	One	One	Four	Four	One	One	Three	None	Three
Library #		146c	YF-Seraseq_WT-30ng1	YF-WT-30ng2b	147c	227	YF-VAF0-40ng	YF-Seraseq_WT-40ng	148c	228	229	YF-VAF0-60ng	231	149c

Suppl. Table S7. Variants detected using wild type (WT) Seraseq ctDNA control. A total of 13 libraries were prepared using the WT control. Different quantities of the WT control were used for library preparation: 30 ng, 40 ng, 50 ng and 60 ng. The number of false positives variants detected in each library are indicated at the bottom of the table. NO indicates that variants were not detected by the PGDx EPR assay. Path, pathogenic; Unc.sig., variants of uncertain significance.

		Seraseq ctDNA controls				SeraSeq Wild type control				Library #
Quantity of Seraseq ctDNA controls for lib. prep.		30 ng	40 ng	50 ng	60 ng	30 ng	40 ng	50 ng	60 ng	
Variants		VAF (%)	(VAF (%)	VAF (%)	VAF (%)	VAF(%)	VAF(%)	VAF(%)	VAF(%)	
EGFR N842K	Unc. Sig.	0.71								118
EGFR N842K	Unc. Sig.	0.58								217
EGFR N842K	Unc. Sig.	0.56								223
EGFR N842K	Unc. Sig.	0.62								120
EGFR N842K	Unc. Sig.		0.54							119
EGFR N842K	Unc. Sig.			0.59						127
EGFR N842K	Unc. Sig.			0.62						224
EGFR N842K	Unc. Sig.				0.54					232
EGFR N842K	Unc. Sig.				0.55					233
KRAS G12C	Path						0.13			YF-VAF0-40ng
KRAS A59V	Path	0.14								218
KRAS A59V	Path		0.1							YF-VAF2-40ng
KRAS A59V	Path			0.1						132
KRAS A59V	Path					0.11				146c
KRAS A59V	Path							0.11		148c
KRAS A59E	Path		0.11							143c
KRAS Q61K	Path	0.12								118
KRAS Q61K	Path							0.21		148c
KRAS A146S	Path			0.13						132
KRAS A146S	Path			0.24						144c
KRAS A146S	Path					0.13				146c
KRAS A146S	Path						0.11			147c
KRAS A146S	Path						0.11			YF-Seraseq_WT-40 ng
KRAS A146S	Path								0.1	YF-VAF0-60ng
ATM S99G	Unc. Sig.		38.72							143c
ATM S99G	Unc. Sig.							36.78		148c
ATM S99G	Unc. Sig.								39.6	149c
ATM D2050_S2058 delinsEEEPYT	Likely Path.				0.46					234
ATM Q2061R fs*34	Likely Path.					0.36				146c
ATM 2262T	Unc. Sig.	0.53								120
ATM G3023C	Unc. Sig.		0.69							YT-LIB19 (VAF05)
NRAS G13C	Path.							0.12		228
NRAS A59D	Path.	0.12								122
NRAS A59D	Path.				0.1					235
NRAS A59D	Path						0.13			YF-Seraseq_WT-40 ng
NRAS A146S	Unc. Sig.							0.1		148c
FGFR1 A343V	Unc. Sig.		0.66							YT-LIB19 (VAF05)
MET A1244D	Unc. Sig.		0.57							YT-LIB19 (VAF05)
BRCA2 E653*	Likely Path.		0.54							136
MYC P17C	Unc. Sig.			0.55						141
BRIPI A267T	Unc. Sig.			0.51						Yinfei7
CDH1 L230I	Unc. Sig.					2.08				YF- WT-30ng
CDH1 A275S	Unc. Sig.							0.57		229
KIT D816Y	Path.					0.16				YF- WT-30ng
RET D850delins ECCIGLFY	Likely Path.						0.22			227
RET L960I	Unc. Sig.								0.78	149c
EZH2 E242*	Likely Path.						0.59			YF-Seraseq_WT-40 ng
RAF Q127K	Unc.sig.						0.59			YF-Seraseq_WT-40 ng
ARID1A Q185H	Unc. Sig.								0.61	YF-VAF0-60ng
BRCA1 W1739C	Path.								0.56	YF-VAF0-60ng
APC E461*	Likely Path.	0.63								223
APC S1346_A1347 insARHKAVEFSSGAKKSPIQH	Unc.sig.								0.28	149c

Suppl. Table S8. False positive variants. False positive (FP) variants were those variants reported by the PGDx EPR software including those variants from the background cell line GM24385 identified as pathogenic, likely pathogenic or variants of uncertain significance; false positive variants for all libraries are shown.

Variant	Lower VAF (%) detected using 50 ng input	Lower VAF (%) detected using 40 ng input	Lower VAF (%) detected using 30 ng input	Lower VAF (%) detected using 60 ng input	Cutoff that PGDx EPR software analysis use for each variant
Single Nucleotide Variants (SNV)					
AKT1 c.49G>A; p. E17K	0.36	0.31	0.48	0.35	~0.31%
APC c.4348C>T; R1450*	0.34	0.36	0.64	0.31	~0.31%
BRAF c.1799T>A; p. V600E	0.16	0.18	0.17	0.17	~0.16%
EGFR c.2369C>T; p. T790M	0.16	0.17	0.19	0.17	~0.16%
EGRF c.2573T>G; p. L858R	0.18	0.16	0.24	0.11	~0.11%
KIT c. 2447A>T; p. D816V	0.11	0.12	0.12	0.18	~0.11%
KRAS c.35G>A; p. G12D	0.15	0.13	0.11	0.12	~0.11%
NRAS c. 182A>G; p. Q61R	0.14	0.14	0.12	0.12	~0.12%
PDGFRA c. 2525A>T; p. D842V	0.13	0.18	0.17	0.1	~0.1%
PIK3CA c. 1633G>A; p. E545K	0.3	0.15	0.19	0.18	~0.15%
PIK3CA c. 3140A>G; p. H1047R	0.1	0.12	0.11	0.17	~0.1%
RET c. 2753T>C; p. M918T	0.11	0.13	0.11	0.1	~0.1%
TP53 c. 524G>A; p. R175H	0.39	0.4	0.37	0.3	~0.3%
TP53 c. 743G>A; p. R248Q	0.39	0.31	0.39	0.3	~0.3%
TP53 c. 818G>A; p. R273H	0.34	0.42	0.31	0.44	~0.31%

Suppl. Table S9. Lower allele frequency detected by PGDx EPR in each SNV from Seraseq ctDNA Reference Material v.2. The cutoff use by PGDx EPR software analysis was determined from the lowest VAF reported from each variant. All these variants are pathogenic and of clinical significance.

Variant	Lower VAF (%) detected using 50 ng input	Lower VAF (%) detected using 40 ng input	Lower VAF (%) detected using 30 ng input	Lower VAF (%) detected using 60 ng input	PGDx EPR software cutoff (VAF%)
**INDELS**					
**APC c. 4666_4667insA; p. T1556fs*3**	**Filter by PGDx software- Insertion in homopolymer 7N**				
**ATM c. 1057_1058delTG; p. C353fs*5**	**0.54**	**0.51**	**0.58**	**0.65**	**~0.54%**
**EGFR c. 2310_2311insGGT; p.D770_N771insG**	**0.19**	**0.12**	**0.16**	**0.13**	**~0.12%**
**EGFR c. 2236_2250del15; p. E746_A750delELREA**	**0.13**	**0.12**	**0.11**	**0.18**	**~0.11%**
**ErbB2 c. 2310_2311insGCATACGTGATG p.E770_A771insAYVM same as: ErbB2 c.2324_2325ins12; p. A775_G776insYVMA**	**0.12**	**0.14**	**0.11**	**0.13**	**~0.11%**
**PDGFRA c. 1694_1695insA; p. S566fs*6**	**1.22**	**0.51**	**0.58**	**0.52**	**~0.51%**
**PIK3CA c.3204_3205insA; p.N1068fs*4**	**0.59**	**0.53**	**0.66**	**0.96**	**~0.53%**
**TP53 c.723delC; p.C242fs*5**	**0.39**	**0.38**	**0.32**	**0.4**	**~0.32%**
**TP53 c.263delC**	**Filter by PGDx software- deletion in homopolymer region 5N>4N**				

Suppl. Table S10. Lower allele frequency detected by PGDx EPR in each indel from Seraseq control using different Seraseq ctDNA inputs to prepare libraries. The cutoff use by PGDx EPR software analysis was determined from the lowest VAF reported from each variant. All these variants are pathogenic except for two highlighted in yellow, which are variants of uncertain significance.

Sample ID	Type of cancer	Stage	Sample from
EW-034 Pre	Colon adenocarcinoma	IV	19-9030
HM-059 Pre	Colon adenocarcinoma	IV	19-9030
HN-008 Pre	Lung adenocarcinoma	IV	19-9030
JR-010 POST	Lung adenocarcinoma, NSCLC, oligometastatic	IV	19-9030
RC-055 (Pre)	Non small cell lung cancer (NSCLC)	IV	19-9030
LD-030	Lung adenocarcinoma, NSCLC	IV	MDL
AS-012	Lung adenocarcinoma; NSCLC	IV	MDL
RW-024	Lung adenocarcinoma	IA	MDL
CK-027	Lung cancer(Squamous cell carcinoma of lung)	IIIB	MDL
JS-040	Non small cell lung cancer (NSCLC)	IV	MDL
JK-049	Non small cell lung cancer (NSCLC)	IV	MDL
GP-051	Non small cell lung cancer (NSCLC)	IIIA	MDL
DK-055	Lung adenocarcinoma, NSCLC	IV	MDL
SH-041	Melanoma of the skin	IV	MDL
MI-043	Melanoma of skin with brain metastases	IV	MDL
AG-042	Esophageal adenocarcinoma	IV	MDL
CS-031 Post	Colon adenocarcinoma	IIIB	19-9030
DH-058	Non small cell lung cancer (NSCLC)	IV	MDL
JM-063	Lung adenocarcinoma	early	MDL
LH-070	Melanoma	IV	MDL
MF-053 Pre	Breast cancer	IV	19-9030
MM-015 Pre	Colon carcinoma	IV	19-9030
CL-080	Lung cancer	IV	MDL
LTW-047 Pre	Lung adenocarcinoma; NSCLC	IVA	19-9030
AD-050 Pre	Colon adenocarcinoma	IV	19-9030
MS-072	Lung adenocarcinoma; NSCLC	IV	MDL
CM-029 Pre	Breast cancer	IV	19-9030
PC-075	Lung squamous cell carcinoma (NSCLC)	IV	MDL
JB-086	Melanoma of skin (hepatocellular carcinoma and breast cancer history)	IV	MDL

Suppl. Table S11. Clinical samples used for validation of EPR assay. Samples were received through the Molecular Diagnostic Laboratory (MDL) or protocol IRB 19-9030.

Gene	Variant (DNA)	Variant (Protein)		Run #8	Run #9	Sample ID
APC	2805C>G	Y935*	Pathogenic	0.54	0.31	SVO-002 POST
KRAS	436G>A	A146T	Pathogenic	1.65	1.3	SVO-002 POST
BRCA2	8259_8265delTCATGGA	h2754Qfs*21	Likely Pathogenic	0.19	0.23	MM-015 Pre
NRAS	181C>A	Q61K	Pathogenic	0.28	0.39	KRS-014 Pre
TP53	524G>A	R175H	Pathogenic	0.83	0.71	MM-015 POST
APC	4326delT	P1443Lfs*30	Pathogenic	0.94	0.88	MM-015 POST
KRAS	34G>T	G12C	Pathogenic	1.62	1.75	EW-034 Pre
KRAS	437C>T	A146V	Pathogenic	1.99	1.64	MM-015 POST
BRAF	1447A>G	K483E	Likely Pathogenic	2.19	1.83	MM-015 POST
TP53	775G>T	D259Y	Pathogenic	3.25	3.1	KRS-014 POST
APC	1690C>T	R564*	Pathogenic	4.44	3.75	KRS-014 POST
TP53	818G>A	R273H	Pathogenic	4.47	4.47	EW-034 POST
KRAS	34G>T	G12C	Pathogenic	5.52	5.47	EW-034 POST
APC	4218_4233delGAGTGAACCATGCAGT	S1407fs*3	Likely Pathogenic	12.6	12.06	EW-034 POST
TP53	775G>T	D259Y	Pathogenic	19.28	19.24	KRS-014 Pre
APC	1690C>T	R564*	Pathogenic	30	28.83	KRS-014 Pre
KRAS	437C>T	A146V	Pathogenic	34.9	39	MM-015 Pre
TP53	818G>A	R273H	Pathogenic	47.39	49.24	EW-034 Pre
APC	4218_4233delGAGTGAACCATGCAGT	S1407fs*3	Likely Pathogenic	51	49	EW-034 Pre
BRAF	1447A>G	K483E	Likely Pathogenic	69	70	MM-015 Pre
TP53	524G>A	R175H	Pathogenic	71.44	71.72	MM-015 Pre
APC	4326delT	P1443Lfs*30	Pathogenic	73.48	75.1	MM-015 Pre

Suppl. Table S12. Data for inter-run reproducibility.

Primer/Probe name	primer/probe sequence	Patient ID#
ALK-E370K-WT	CCACAAC+G+AGGCTGC	EW-034
ALK-E370K-MUT	CCACAA+C+A+AGGCTGC	EW-034
ALK-E370K-F	AGCCCTCTGGAAGGTACA	EW-034
ALK-E370K-R	AGAAGTACTTACCCATGCTTCC	EW-034
KRAS-G12C-WT	CGCCAC+C+AGCTCC	EW-034
KRAS-G12C-MUT	ACGCCA+C+A+AGCTCC	EW-034
TP53-R273H	DMH0000094 (Qiagen)	EW-034
TP53-R273H	DMH0000094 (Qiagen)	EW-034
TP53-R273H	DMH0000094 (Qiagen)	EW-034
TP53-R273H	DMH0000094 (Qiagen)	EW-034
APC- S1407fs- WT	ACTGC+A+TGG+T+TCACTC	EW-034
APC- S1407fs-MUT-PGDx	CCAT+TC+C+TG+A+ACGGA	EW-034
APC- S1407fs --MUT-Tempus	CCAT+TC+C+C+TGAACGG	EW-034
APC- S1407fs-F	TTGAGAGTCGTTCGATTGCCAGCT	EW-034
APC- S1407fs -R	CATGGTTTGTCCAGGGCTAT	EW-034
ALK-A1200V-F	ACATTGTTCGCTGCATTGG	SH-041
ALK-A1200V-R	TCTCGGAGGAAGGACTTGA	SH-041
ALK-A1200V-WT	CTCATG+G+C+GGGGG	SH-041
ALK-A1200V-MUT	CTCATG+G+T+GGGGG	SH-041
BRAF-V600E	DMH0000004 (Qiagen)	SH-041
BRAF-V600E	DMH0000004 (Qiagen)	SH-041
BRAF-V600E	DMH0000004 (Qiagen)	SH-041
BRAF-V600E	DMH0000004 (Qiagen)	SH-041
BRAFG265R-WT	T+T+A+TA+A+C+C+AC+A+TG	LTW-047
BRAFG265R-MUT	T+T+A+TA+A+C+G+AC+A+TG	LTW-047
BRAF-K483E-WT	CAACA+TT+T+T+CACTGCCA	MM-015
BRAF-K483E-Mut	CAACATT+T+C+CACTGCCA	MM-015
BRAF-K483E-F	CACTTGGAGTAACAATTGCCTTT	MM-015
BRAF-K483E-R	CAAGCTCACCTGAGTACTCCTA	MM-015
KRAS-A146V-WT	ACA+TCA+G+C+AA+AGACAAG	MM-015
KRAS-A146V-MUT	ACA+TCA+G+T+AA+AG+ACAAG	MM-015
KRAS-A146V-F1	CTCAGGACTTAGCAAGAAG	MM-015
KRAS-A146V-R1	CTGTATTTATTTCAGTGTTACTTACC	MM-015
TP53-R175H-F	CTGTACCACCATCCACTACAAC	MM-015
TP53-R175H-R	TCCTGACCTGGAGTCTTCC	MM-015
TP53-R175H-WT	CATGGG+C+G+GCATGAA	MM-015
TP53-R175H-MUT	CATGGG+C+C+GCATGAA	MM-015
APC-P1443Lfs*30-F	CCCAGTGATCTTCCAGATAGC	MM-015
APC9-P1443Lfs*30-R	TAGGTACTTCTCGCTTGGTTTG	MM-015
APC-P1443Lfs*30-WT	CACCAC+C+T+CC+TCAAA	MM-015
APC-P1443Lfs*30-MUT	CACCA+C+C+CC+TCAAA	MM-015
EGFR E746_S752delinsA-WT1	A+A+TT+A+AG+AG+A+AGC+A+AC	AS-012
EGFR E746_S752delinsA- MUT1	CGCTATCA+AGGC+TCCG+AAAG	AS-012
EGFR S752F- F1	CCCGTCGCTATCAAGGAATTA	AS-012
EGFR S752-R1	CCACACAGCAAAGCAGAAAC	AS-012
PIK3CA E726K-WT1	AAG+A+AGGA+T+G+AA+AC+ACAA	AS-012
PIK3CA E726K-MUT1	AAG+A+A+GGA+T+A+AA+A+C+ACAA	AS-012
EGFR-E746_A750del-F	GGATCCCAGAAGGTGAGAAAG	HN-008
EGFR-E746_A750del-R	CAGCAAAGCAGAAACTCACATC	HN-008
TP53-N239D	CAGTGTGATGATGGTGAGGATG	HN-008
TP53-N239D	CATG+TG+T+A+AC+AG+TTCC	HN-008
TP53-N239D	CATGTG+T+G+ACAG+TTCC	HN-008
TP53-N239D	TATCTCCTAGGTTGGCTCTGAC	HN-008
KRAS-G12A-MUT	AGCT+G+C+TGGCGTAG	JS-040
KRAS-G12A-WT	AGCT+G+G+TGGCGTA	JS-040
KRAS-G12A-F	GACTGAATATAAACTTGTGGTAGTTGG	JS-040
KRAS-G12A-R	GATTCTGAATTAGCTGTATCGTCAAG	JS-040
TP53-G245C-F	TATCTCCTAGGTTGGCTCTGAC	DK-055
TP53-G245C-R	CAGTGTGATGATGGTGAGGATG	DK-055
TP53-G245C-WT	CATGGG+C+GGCATGAA	DK-055
TP53-G245C-MUT	CATGGG+C+T+GCATGAA	DK-055
BRAF- V600K-F	TTCATGAAGACCTCACAGTAAA	MI-043
BRAF-V600K-R	GGATCCAGACAACTGTTCAAA	MI-043
BRAF- V600K-WT	TAGCT+AC+A+G+TG+AAATCTC	MI-043
BRAF-V600K-MUT	AGCT+AC+A+A+AG+AAATCTCG	MI-043
PIK3CA E542K-WT1	CCTCTC+TC+T+G+AA+A+TCACTG	HM-059
PIK3CA E542K-MUT1	CC+TC+TC+TC+T+A+AA+A+TC+ACTG	HM-059

Suppl. Table S13. Primers/probes used for dPCR.

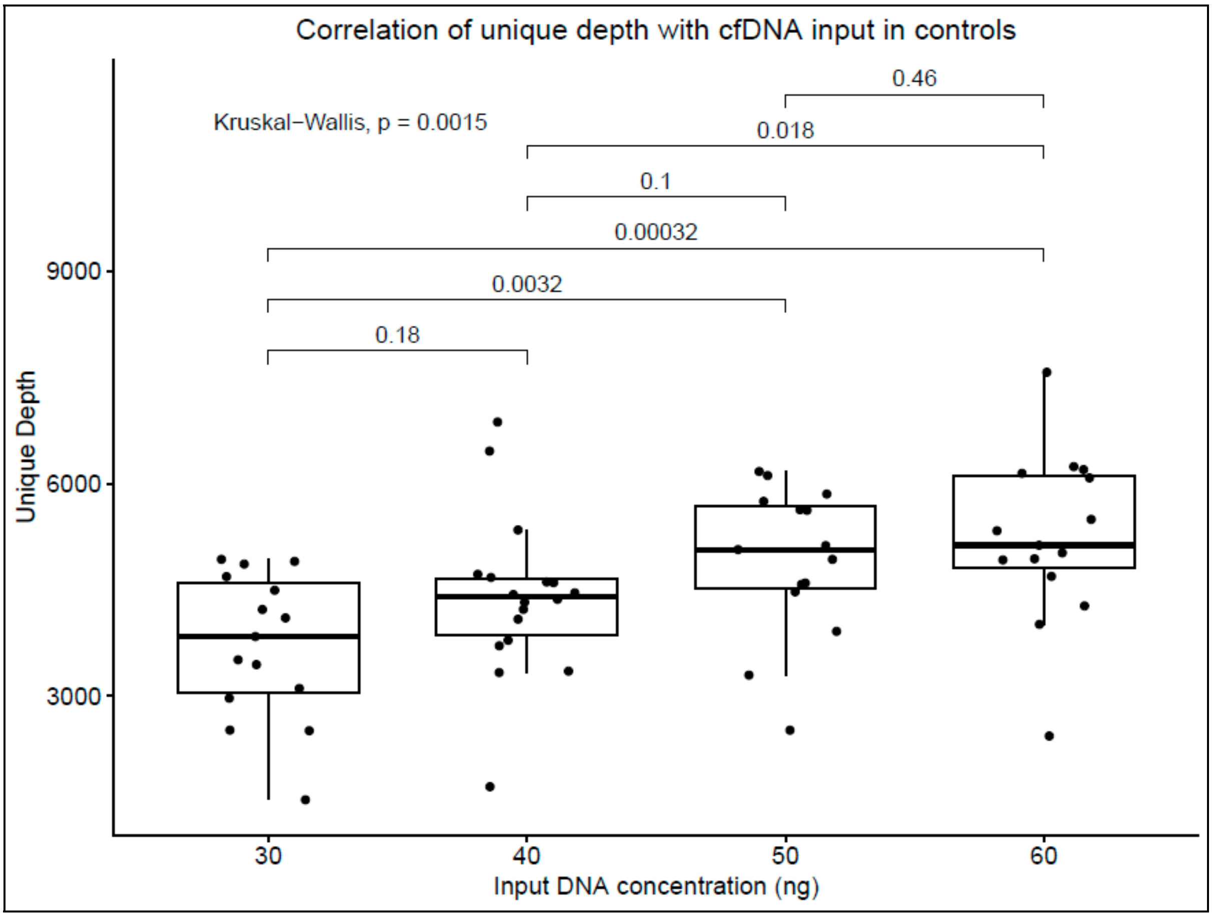

Suppl. Figure S1. Unique depth using different quantities of ctDNA SeraSeq Reference Material v.2

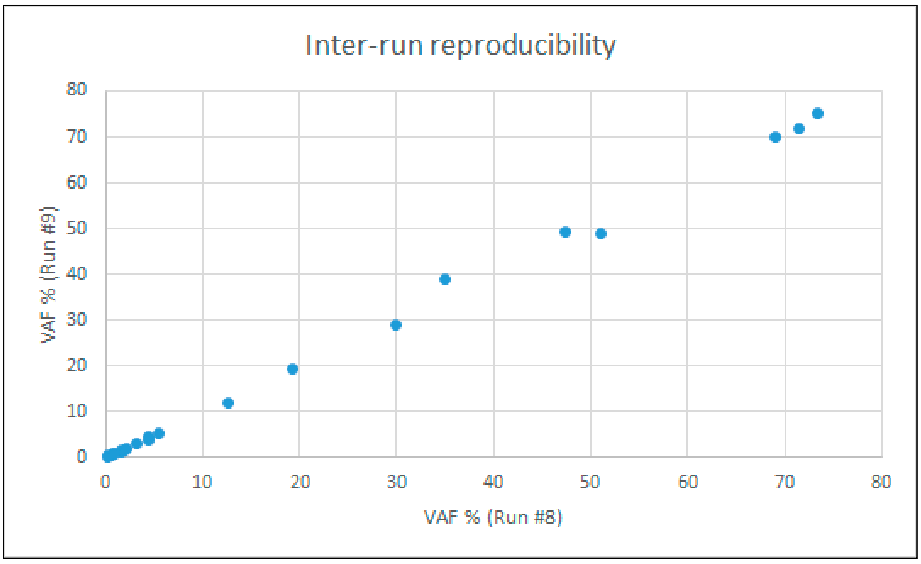

Suppl. Figure S2. Inter-run reproducibility. The VAFs of the variants detected from same libraries run twice (Run #8 vs. Run #9) were compared. Equation: Y = 1.016*X - 0.1825; R2= 0.9982; p<0.0001.

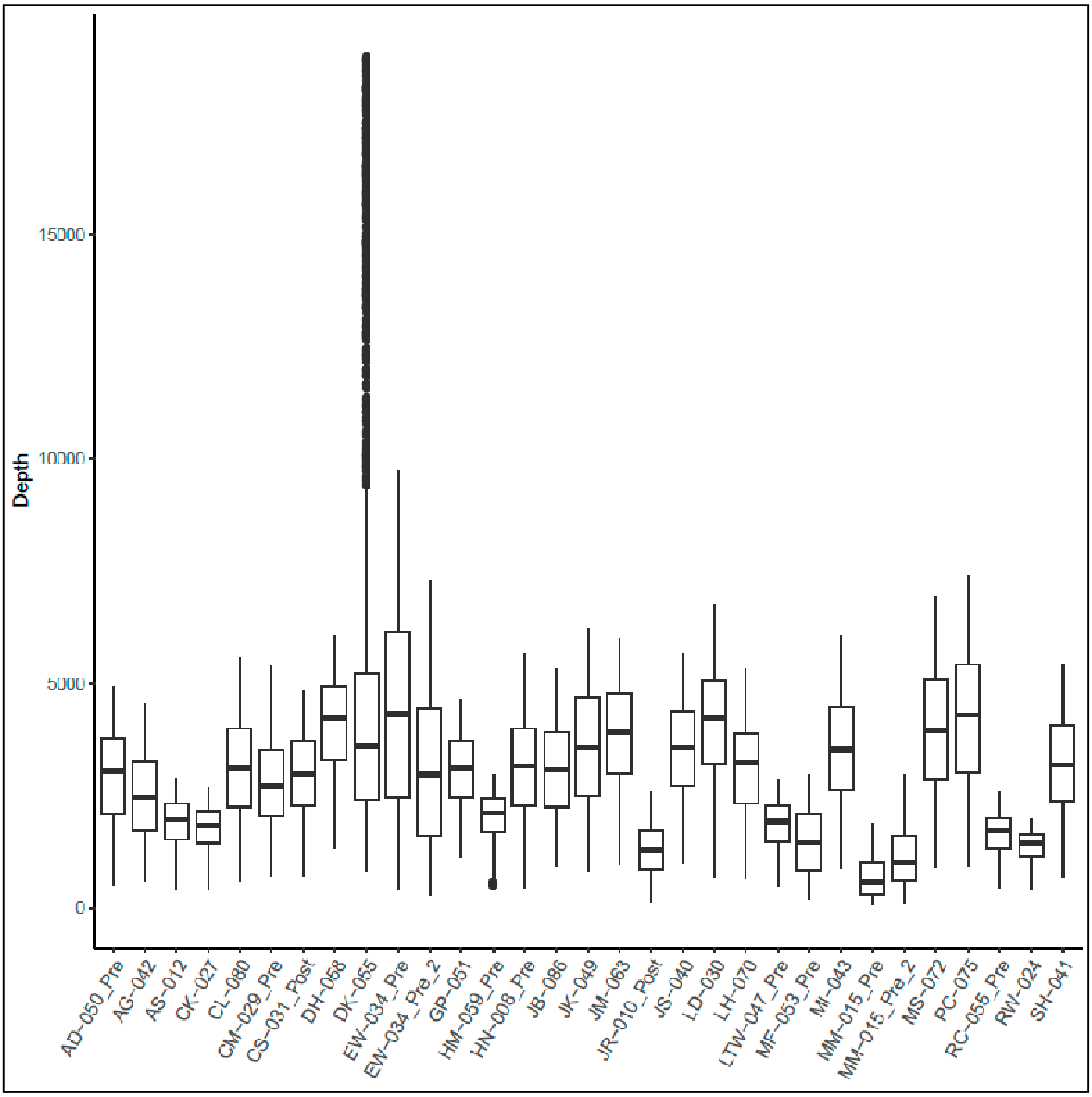

Suppl. Figure S3. Depth distribution for each library from clinical samples. cfDNA libraries from clinical samples are indicated in the x axis and the depth reads in each library is indicated in the y axis.
